# Hepatic Branch Vagotomy Modulates the Gut-Liver-Brain Axis in Murine Cirrhosis

**DOI:** 10.3389/fphys.2021.702646

**Published:** 2021-06-25

**Authors:** Yuan Zhang, Jason D. Kang, Derrick Zhao, Siddartha S. Ghosh, Yanyan Wang, Yunling Tai, Javier Gonzalez-Maeso, Masoumeh Sikaroodi, Patrick M. Gillevet, H. Robert Lippman, Phillip B. Hylemon, Huiping Zhou, Jasmohan S. Bajaj

**Affiliations:** ^1^Division of Microbiology and Immunology, Central Virginia Veterans Health Care System, Virginia Commonwealth University, Richmond, VA, United States; ^2^Division of Nephrology, Virginia Commonwealth University, Richmond, VA, United States; ^3^Department of Physiology and Biophysics, Virginia Commonwealth University, Richmond, VA, United States; ^4^Microbiome Analysis Center, George Mason University, Manassas, VA, United States; ^5^Department of Pathology, Central Virginia Veterans Health Care System, Richmond, VA, United States; ^6^Division of Gastroenterology, Hepatology, and Nutrition, Central Virginia Veterans Health Care System, Virginia Commonwealth University, Richmond, VA, United States

**Keywords:** vagotomy, pathobiont, inflammation, BDNF, hepatic encephalopathy, microbiota (16S)

## Abstract

**Background:**

Cirrhosis and hepatic encephalopathy (HE) are linked with an altered gut-liver-brain axis, however, the relative contribution of hepatic vagal innervation is unclear. We aimed to determine the impact of hepatic vagotomy on the gut microbiome, brain, and liver in murine cirrhosis.

**Methods:**

10–15-week-old male C57BL/6 mice with and without hepatic vagotomy underwent carbon tetrachloride (CCl4) gavage for 8 weeks. Frontal cortex [inflammation, glial/microglial activation, BDNF (brain-derived neurotrophic factor)], liver [histology including inflammation and steatosis, fatty acid synthesis (sterol-responsive binding protein-1) SREBP-1, insulin-induced gene-2 (Insig2) and BDNF], and colonic mucosal microbiota (16srRNA microbial sequencing) were evaluated on sacrifice. Conventional mice with and without cirrhosis were compared to vagotomized counterparts.

**Results:**

*Conventional control vs. cirrhosis*: Cirrhosis resulted in dysbiosis, hepatic/neuro-inflammation with glial/microglial activation, and low brain BDNF vs. controls. *Conventional control vs. vagotomy controls:* Vagotomized control mice had a lower colonic dysbiosis than conventional mice but the rest of the hepatic/brain parameters were similar. *Conventional cirrhosis vs. vagotomized cirrhosis:* After vagotomy + cirrhosis, we found lower dysbiosis but continuing neuroinflammation in the absence of glial/microglial activation vs. conventional cirrhosis. Vagotomy + Cirrhosis groups showed higher hepatic steatosis due to higher SREBP1 and low Insig2 protein and altered activation of key genes involved in hepatic lipid metabolism and inflammation. BDNF levels in the brain were higher but low in the liver in vagotomy + cirrhosis, likely a protective mechanism.

**Conclusions:**

Hepatic vagal innervation affects the gut microbial composition, hepatic inflammation and steatosis, and cortical inflammation and BDNF expression and could be a critical modulator of the gut-liver-brain axis with consequences for HE development.

## Introduction

The gut-liver-brain axis mediates the development and progression of complications of cirrhosis, such as hepatic encephalopathy (HE) (Kang et al., 2016a; Ochoa-Sanchez and Rose, 2018). Most therapies for HE are focused on the modulation of the gut microbial milieu using laxatives and antibiotics (Vilstrup et al., 2014). In addition to the microbial metabolite and barrier impairment, gut-brain axis alterations could also be modulated through vagal innervation (Cryan et al., 2019). Prior murine studies have shown that transplanted stool from patients with cirrhosis, but not from healthy controls, results in neuro-inflammation in germ-free mice (Liu et al., 2020). In cirrhosis, the additional impact of hepatic failure and inflammation adds to the overall inflammatory milieu that can affect brain function (Shawcross et al., 2004, 2011). The hepatic branch of the vagus is associated with modulating metabolic signals between the brain and the liver (Pocai et al., 2005; Harada et al., 2014; Metz and Pavlov, 2018). The liver-brain connection through the hepatic vagus branch could be used to determine the relative contribution of the liver-brain aspect of the gut-liver-brain axis.

Our aim was to evaluate the effect of isolated hepatic vagotomy on gut microbiota, liver inflammation, and brain inflammation in the setting of murine cirrhosis.

## Materials and Methods

**FIGURE 1:**
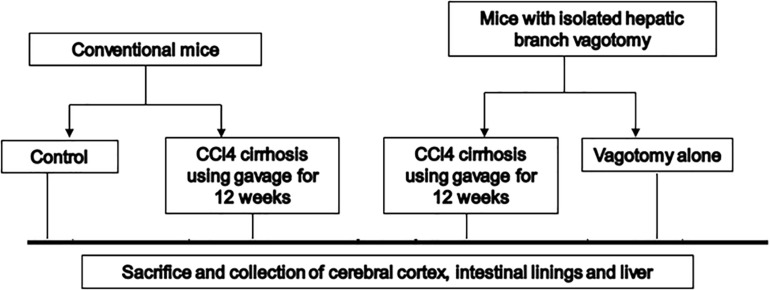
Schema of the experiment. CCl4, carbon tetrachloride.

We used 10–12-week-old C57BL/6 male mice for this experiment (Figure 1). Cirrhosis was induced in a subgroup of mice using our protocol of CCl4 gavage for 14 weeks (Kang et al., 2016a). Another group of 10–12-week-old male C57BL/6 mice with isolated hepatic branch vagotomy were obtained from Charles River laboratories. After acclimatization at the VCU animal facility, the mice were then again divided into those followed as controls and those who received 14 weeks of CCL4 gavage. All mice were sacrificed at week 14 (*n* = 6 per group). At the time of sacrifice, we collected the small intestinal mucosa, large intestinal mucosa, liver, and frontal cortices from all mice. In the brain, we studied messenger RNA (mRNA) expression of (a) neuroinflammation [interleukin (IL)-1β, monocyte chemoattractant protein 1 (MCP1)]; (b) glial/microglial activation [ionized calcium-binding adaptor molecule 1 (IBA1) and glial fibrillary acidic protein (GFAP)]; (c) brain regeneration and plasticity [brain-derived neurotrophic factor (BDNF)]. The liver tissues were processed for histological analysis and isolation of total RNA and total protein lysate. The mRNA levels of the key genes involved in hepatic lipid metabolism and inflammation were measured by real-time quantitative PCR (RT-qPCR) (Table 1). The protein levels of SREBP-1 (Purified Mouse Anti-SREBP-1, from BD Pharmingen, Cat#557036) and Insig2 (Santa Cruz, Cat# sc-34823). Histological analysis of the hepatic inflammation, steatosis, fibrosis or cirrhosis was done by a pathologist in a blinded fashion with hematoxylin and eosin and trichrome staining using the NASH-Clinical Research Network criteria.

**TABLE 1:** Real-time PCR Primers.

Name of gene	Genebank number	Forward primer	Reverse primer
BDNF	X55573	ACGGTCACAGTCCTAGAG	AGCCTTCCTTGGTGTAAC
Cd11b	NM_008401	CGGTAGCATCAACAACAT	GCATCAAAGAGAACAAGGT
Ces1g	NM_021456	CGCCAGAAGACTGAAGATGAGC	TGGTGCCTTTGGCAGCAACACT
Ces2	NM_133960.5	GCTCTCCAAGTGGCACATTTCC	CAAAGGCAACGTCATCACCATGG
CK-19	NM_008471	CGAGATTACAACCACTAC	GTTCTGTCTCAAACTTGG
Col1αa	NM_007742.4	ACATGTTCAGCTTTGTGGACC	TAGGCCATTGTGTATGCAGC
GFAP	NM_001131020	CAACCTGGCTGCGTATAG	CGAACTTCCTCCTCATAGAT
IBA-1	AB036423.1	GTCCTTGAAGCGAATGCTGG	CATTCTCAAGATGGCAGATC
IL-1b	NM_008361.4	GCAACTGTTCCTGAACTCAACT	GCCCAACATCTTTTGGGGTCCGTCAACT
LPL	NM_008509	GTCTAACTGCCACTTCAACC	CACCCAACTCTCATACATTCC
MCP-1	NM_011333	CTTCTGGGCCTGCTGTTCA	CCAGCCTACTCATTGGGATCA
Sirt1	NM_019812	TAGCACTAATTCCAAGTTCTATAC	TAACATCGCAGTCTCCAA
α-SMA	NM_007392.3	TGAAGAGCATCCGACACT	AGCCTGAATAGCCACATAC

Large intestinal mucosal microbiota was analyzed using 16S ribosomal RNA (rRNA) sequencing after DNA was extracted (Gillevet et al., 2010).

Statistical analysis: We analyzed continuous data using unpaired *t*-tests/ANOVA for mean and SEM and Kruskal–Wallis/Mann–Whitney for comparing medians. For *in vivo* studies, in order to determine the group size, we will perform the power calculations to detect a 25% difference at a power of 0.8 and a confidence level of 95% for neuro-inflammation. Based on the data from an earlier mouse study with the standard deviation of 50% for each group, the group sizes equal to or greater than 6 are required. This was also based on our prior study of changes in gut microbiome affecting neuro-inflammation (Liu et al., 2020). Data are presented as mean ± SEM unless otherwise specified. We compared changes in microbial composition between mouse groups using linear discriminant effect size (LEfSe) (Segata et al., 2011) analysis.

Approvals were obtained from the Institutional Animal Care and Use Committee at VCU before study initiation. All animals received humane care according to the criteria outlined in the Guide for the Care and Use of Laboratory Animals. All authors had access to the study data and had reviewed and approved the final manuscript.

## Results

There were no obvious changes in health appearance, weight changes or sickness behavior noted in any of the mice before the sacrifice.

### Frontal Cortex Evaluation

#### Neuro-Inflammation

**FIGURE 2:**
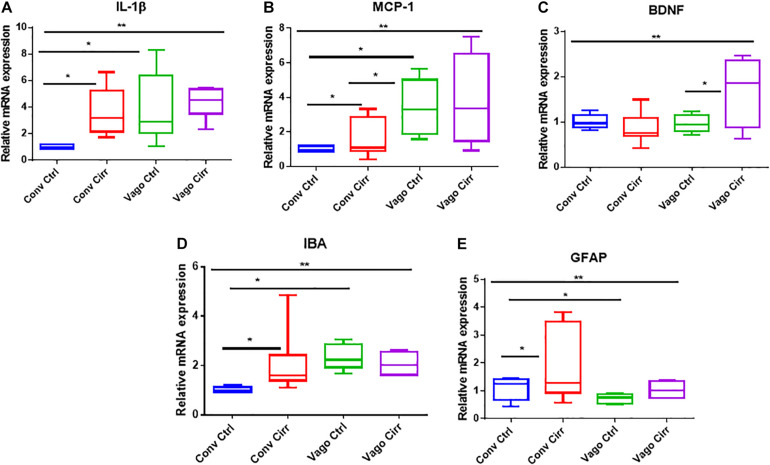
mRNA expression in frontal cortices presented as median and 95% CI. **(A)** Interleukin 1 β, **(B)** Monocyte chemoattactant protein 1, **(C)** Glial fibrillary acidic protein and **(D)** ionized calcium-binding adaptor molecule 1. **(E)** Brain-derived neurotrophic factor. Conv, conventional; Ctrl, control; Vago-Ctrl, isolated hepatic branch vagotomy mice; Vago-Cirr, mice with CCl4 cirrhosis after isolated hepatic branch vagotomy. *p < 0.05–0.01, **p < 0.01–0.001, and ***p < 0.001 on Mann–Whitney or Kruskal–Wallis as appropriate.

Two neuro-inflammatory markers (IL-6 and MCP-1) were significantly higher in conventional cirrhotic mice than controls (Figure 2). There was a significant increase in IL-1β and MCP-1 mRNA expression in mice with vagotomy compared to the controls, which marginally changed after the development of cirrhosis. Glial (GFAP) and microglial (IBA) markers were higher in cirrhotic mice without vagotomy. However, these changes were largely abrogated after vagotomy. BDNF (Cattaneo et al., 2016) was not affected by cirrhosis in the conventional state. Vagotomized mice showed increased frontal cortex expression and BDNF.

### Mucosal Microbial Changes

**FIGURE 3:**
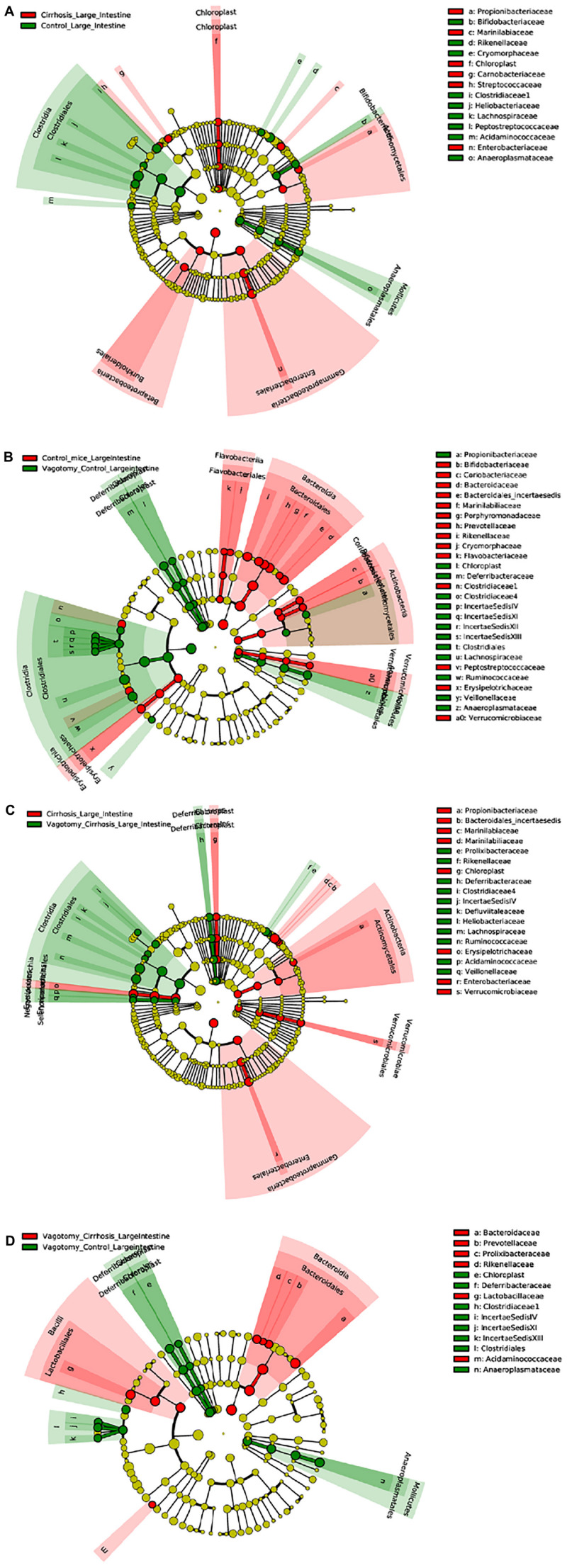
Linear discriminant analysis Effect Size (LEfSe) cladograms of large intestinal mucosal comparisons. Significant differences are color coded. **(A)** Conventional controls vs. conventional cirrhosis. **(B)** Conventional controls vs. vagotomized controls. **(C)** Conventional cirrhosis vs. vagotomized cirrhosis. **(D)** Vagotomized controls vs. vagotomized cirrhosis

An overview of microbial comparisons are shown in Table 2 and Figure 3.

**TABLE 2:** LEfSe changes in colonic mucosal microbiota in Conventional mice and mice with Vagotomy.

LEfSe comparison	Higher in controls	Higher in cirrhosis
Control vs. Cirrhosis	Actinobacteria_Bifidobacteriaceae	Actinobacteria _Proprionibacteriaceae
	Bacteroidetes_Cryomorphaceae	Bacteroidetes_Marinilabiliaceae
	Bacteroidetes_Rikenellaceae	Cyanobacteri a_Chloroplast
	Firmicutes_Acidaminococcaceae	Firmicutes_St reptococcaceae
	Firmicutes_Heliobacteriaceae	Proteobacteri a_Enterobacteriaceae
	Firmicutes_Lachnospiraceae	Proteobacteri a_Burkholderiaceae
	Firmicutes_Peptostreptococcaceae	
	Tenericutes_Anaeroplasmataceae	
	**Higher in controls**	**Higher in vagotomy controls**
Control vs. Vagotomy control	Proteobacteria_Enterobacteriaceae	Firmicutes_Veillonellaceae
	Firmicutes_Erysipelothricaceae	Firmicutes_Lachnospiracae
	Firmicutes_Peptostreptococcaceae	Firmicutes_Ruminococcaceae
	Actinobacteria_Corynebacteriaceae	Firmicutes_ClostridialesIncSedXI
	Actinobacteria_Bifidobacteriaceae	Firmicutes_ClostridialesIncSedIV
	Actinobacteria_Coriobacteriacae	Actinobacteria_Propionibacteriaceae
	Bacteroidetes_Bacteroidaceae	Tenericutes_Aneroplasmataceae
	Bacteroidetes_Prevotellaceae	
	Bacteroidetes_Cryomorphaceae	
	Bacteroidetes_Marinilibiaceae	
	Bacteroidetes_Flammeovirgiaceae	
	Bacteroidetes_Flavobacteriaceae	
	Bacteroidetes_Porphyromonadaceae Verrucomicrobia_Verrucomicrobiaceae	
	**Higher in cirrhosis**	**Higher in vagotomy cirrhosis**
Cirrhosis vs. Vagotomy Cirrhosis	Cyanobacteria_Chloroplast	Bacteroidetes_Rikenellaceae
	Actinobacteria_Propionibacteriaceae	Bacteroidetes_Prolixibacteriaceae
	Bacteroidetes_Bacteroidaceae	Deferribacters_Deferribacteriaceae
	Bacteroidetes_Marinilibilaceae	Firmicutes_Lachnospiracae
	Firmicutes_Erysipelothricaceae	Firmicutes_Ruminococcaceae
	Proteobacteria_Enterobacteriaceae	Firmicutes_Veillonellaceae
		Firmicutes_Heliobacteriaceae
		Firmicutes_Acidaminococcaceae
		Firmicutes_Clostridiaceae
		Firmicutes_Defluviitaleaceae

*Microbiota presented as Phylum_Family. LEfSe: Linear Discriminant Analysis Effect Size.*

#### Conventional Controls vs. Conventional Cirrhosis Comparisons

As expected, conventional controls had higher potentially beneficial taxa such as Lachnospiraceae and Bifidobacteriaceae compared to conventional mice with cirrhosis that demonstrated greater pathobionts such as Enterobacteriaceae and Streptococcaceae.

#### Conventional Controls vs. Vagotomized Control Comparisons

After vagotomy, control mice had greater beneficial taxa belonging to Firmicutes, including Lachnospiraceae, Ruminococcaceae and Rikenellaceae, and lesser relative abundance of Enterobacteriaceae and Verrucomicrobiaceae compared to conventional control mice.

#### Conventional Cirrhosis vs. Vagotomy Cirrhosis Comparisons

Mice with cirrhosis after vagotomy had a higher representation of beneficial Firmicutes families compared to conventional cirrhosis.

### Liver Findings

#### Histological Analysis

**FIGURE 4:**
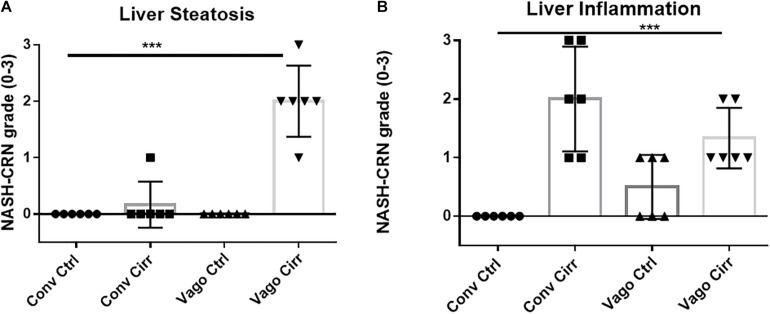
Histological evaluation of steatosis and inflammation according to NASH-Clinical Research Network criteria. Conv, conventional; Ctrl, control; Cirr, CCL4 cirrhosis; VagoCtrl, isolated hepatic branch vagotomy mice; VagoCirr, Mice with CCl4 cirrhosis after isolated hepatic branch vagotomy. ***p < 0.001 on Kruskal–Wallis Individual mouse data and median 95% CI are presented. **(A)** Steatosis grade. **(B)** Inflammatory grade.

**FIGURE 5:**
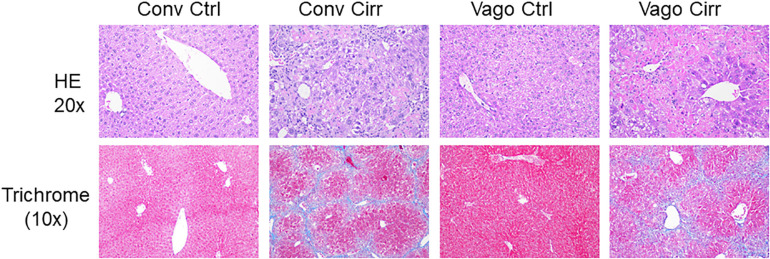
Representative liver histological sections in H/E 20X and trichrome 10X showing normal histology in both control groups, cirrhosis on trichrome in both cirrhosis groups and steatosis in Vagotomized cirrhosis liver. Conv, conventional; Ctrl, control; Cirr, CCL4 cirrhosis; VagoCtrl, isolated hepatic branch vagotomy mice; VagoCirr, Mice with CCl4 cirrhosis after isolated hepatic branch vagotomy.

As expected, mice receiving CCl4 for 12 weeks developed cirrhosis on histology regardless of vagotomy. No changes in histology were seen at 12 weeks between conventional control and control vagotomized mice (Figures 4, 5). The inflammatory grade increased as expected with cirrhosis development, was lower in the vagotomized group, but the opposite was seen with the steatosis grade.

#### Liver Inflammation and Fibrosis

**FIGURE 6:**
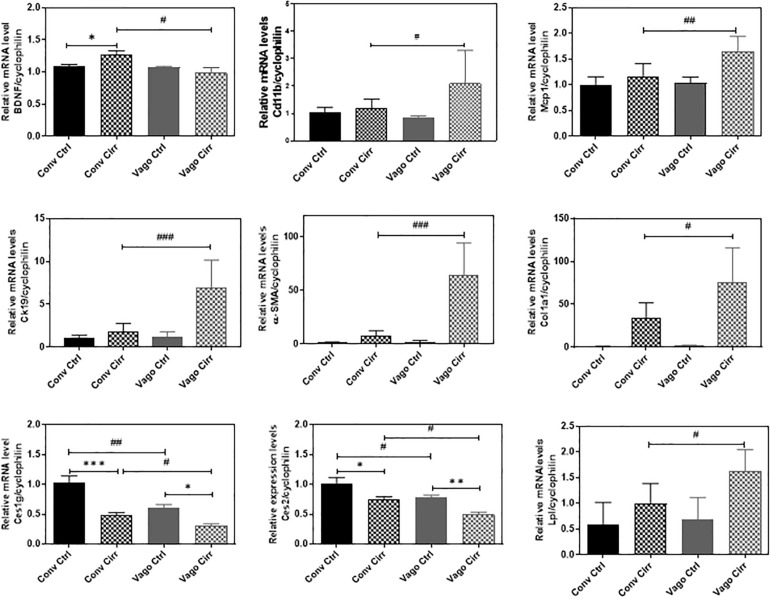
Hepatic Expression of BDNF and Genes involved in fatty acid metabolism and inflammation presented as Mean ± SEM. Conv, conventional; Ctrl, control; Cirr, CCL4 cirrhosis; VagoCtrl, isolated hepatic branch vagotomy mice; VagoCirr, mice with CCl4 cirrhosis after isolated hepatic branch vagotomy. Comparisons using Mann–Whitney test, ^#^/*p < 0.05, ^##^/**p < 0.01, and ^###^/***p < 0.0001.

In addition to the histological inflammatory grade, there was a significant increase in mRNA expression of MCP-1, Cd112b, and CD63 in the mice that developed cirrhosis in the setting of vagotomy (Figure 6). BDNF expression increased in conventional mice with cirrhosis but not in vagotomized ones. In addition, the mRNA levels of Ck-19, α-SMA and Col1α were significantly upregulated in vagotomized cirrhotic mice.

#### Steatosis

Given the increase in steatosis with vagotomy cirrhotic mice, we examined the key genes involved in hepatic lipid metabolism using real-time RT-PCR and Western blot analyses. The RT-PCR further showed the upregulation of lipoprotein lipase (LPL) and downregulation of carboxylesterases, Ces1g and Ces2.

**FIGURE 7:**
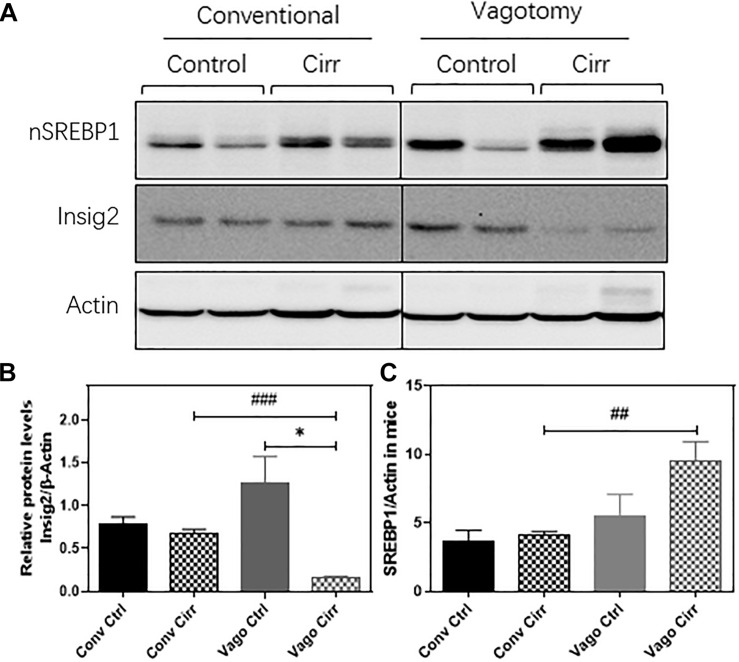
Western Blot expression of Hepatic SREBP1 and Insig2 using Actin as the reference protein. **(A)** gels, **(B)** Mean ± SEM for SREBP1/Actin ratio, **(C)** Mean ± SEM for Insig2/Actin Ratio. Conv, conventional; Ctrl, control; Cirr, CCL4 cirrhosis; VagoCtrl, isolated hepatic branch vagotomy mice; VagoCirr, mice with CCl4 cirrhosis after isolated hepatic branch vagotomy. ^###^*p* < 0.001 and ^##^*p* < 0.01 between Conv Cirr and Vago Cirr, *p < 0.05 between VagoCtrl and VagoCirr on Mann–Whitney tests.

As shown in Figure 7, the nuclear form of SREBP1 protein levels were significantly increased in the vagotomy-cirrhosis group compared to the conventional cirrhosis group. Furthermore, the protein level of Insig 2 was significantly reduced in vagotomy cirrhotic mice.

## Discussion

Connections between the gut, liver, and brain are germane toward the development and progression of cirrhosis, but the role of hepatic vagal output requires greater clarification. Using a mouse model of hepatic branch vagotomy, we found that vagal output from the liver is associated with changes in gut microbial composition, neuro-inflammation, and plasticity, as well as hepatic steatosis and inflammation. These results demonstrate that hepatic vagal innervation can affect the gut-liver-brain axis in cirrhosis.

Although previous findings suggested that the hepatic branch of the vagus has a pivotal role in transmitting information from the liver to the brain, how this modulates the effects of cirrhosis remained largely unknown (Pocai et al., 2005; Harada et al., 2014). Mice with vagotomy showed lower dysbiosis with a higher relative abundance of potential autochthonous taxa such as Lachnospiraceaeae and Ruminococcaceae regardless of whether this was controls or in mice that developed cirrhosis. This interesting role of the hepatic vagus adds another dimension to the gut-liver axis in addition to the previously described factors such as intestinal barrier integrity, bile acid features and bacterial products. The data show that vagal output from the liver enhances the overgrowth of potential pathobionts since vagotomy reduced dysbiosis in the mucosal microbiome. This is even more striking because hepatic branch vagotomy does not affect gastric acid secretion, intestinal motility or physical connections between the liver and the gut.

While the findings of lower dysbiosis after vagotomy seem to be at odds with the higher brain inflammation and higher liver steatosis, most of these changes only occurred after CCl4 gavage and were associated with differential changes in BDNF expression. However, some microbial taxa belonging to Ruminococcaceae that are typically lower in advancing cirrhosis, are actually associated with fibrosis and metabolic syndrome in patients with liver steatosis (Boursier et al., 2016; Lee et al., 2020). In addition, Verrucomicrobiaceae, which includes the beneficial taxon, *Akkermansia muciniphilia* were lower in vagotomized controls, which could also promote hepatic steatosis (Everard et al., 2013). Following this, we found higher hepatic steatosis in vagotomized cirrhotic mice, which had higher mucosal Ruminococcaceae. With the appearance of cirrhosis, these changes in Verrucomicrobia were not seen, likely due to the cirrhosis state reducing these organisms uniformly regardless of vagotomy.

Prior studies in vagotomized mice in the setting of NAFLD before cirrhosis have shown greater steatosis, but we extended these in a cirrhosis model of CCl4 gavage that typically does not demonstrate steatosis. While the exact mechanisms are unclear, there was an upregulation of the activated nuclear form of SREBP1 (nSREBP1) protein level in the liver after vagotomy. This increase in nSREBP1 was associated with a relative loss of Insig2 after vagotomy in the liver. Insig2 is an ER stress-responsive gene, which prevents the proteolytic processing of SREBP-1c from forming a maturing form, a critical transcriptional regulator of hepatic fatty acid metabolism (Takaishi et al., 2004). LPL is an important player in regulating lipid metabolism and energy balance. The upregulation of LPL has been reported to exacerbate liver fibrosis (Teratani et al., 2019). The carboxylesterases plays a critical role in hydrolyze a variety of xenobiotic and endogenous compounds and including lipid esters. Six human CES genes have been identified. CES1 and CES2 are the two most prominent genes, which are mainly expressed in the gastrointestinal tract and liver. In mice, eight genes belong to Ces1 have been identified with relatively unique tissue expression patterns. Compared to other Ces1 family members, Ces1g is highly expressed in the liver and intestine (Lian et al., 2018). Previous studies have shown that deficiency of Ces1g or Ces2a was linked to metabolic diseases. It has been reported that that Ces1g suppresses the activity of Srebp1c promoter and enhances the degradation of Srebp1 (Xu et al., 2001). In addition, Ces1g inhibits Insig 1 degradation and *de novo* lipogenesis. Downregulation of Ces1g may attribute to activation of SREBP1c, leading to lipogenesis. Liver specific expression of Ces1g reduces hepatic steatosis (Bahitham et al., 2016). In addition, downregulation of Ces2 is associated with NASH disease progression and high-fat-diet-induced steatosis (Li et al., 2016). Hepatic CES2 plays a key role in fatty acid oxidation and inhibiting lipogenesis. We also found that the expression of Ces2 was downregulated in both conventional and vagotomy cirrhotic mice and vagotomy further inhibited Ces2 expression. Therefore, these could be the potential mechanisms behind the development of steatosis in the liver and lower inflammation after vagotomy and cirrhosis compared to the conventional animals (Gao et al., 2015; Amir et al., 2020).

Despite the increase in Ruminococcaceae in vagotomized animals that can promote steatosis, vagotomized animals did have higher relative abundances of other potentially beneficial taxa such as Lachnospiraceae and reduction in pathobionts like Enterobacteriaceae (Kang et al., 2016a,b, 2017). In prior studies of germ-free mice colonized with stools from differing human phenotypes, there was an increase in hepatic and frontal cortical neuro-inflammation in mice that received stool from patients with cirrhosis (Liu et al., 2020). Other studies demonstrated that susceptibility to alcohol-related liver disease was also modulated by the donor of the microbiota, whether it be a different mouse group or humans (Llopis et al., 2016; Cassard et al., 2017). Microglial and glial activation are usually associated with cirrhosis-related neuro-inflammation but in vagotomized mice, the expression of GFAP and IBA were abrogated even with cirrhosis. Therefore, factors other than dysbiosis and glial/microglial activation, such as systemic inflammation and BDNF may be associated with neuroinflammation in mice with hepatic branch vagotomy and cirrhosis (Shawcross et al., 2011; Gorg et al., 2015). BDNF is usually thought of as a primary neurotrophic molecule but of late studies have shown it to be an important part of the liver-brain axis (Pocai et al., 2005; Bercik et al., 2011; Cattaneo et al., 2016). The role of BDNF is complex since it can modulate insulin resistance and liver disease in animal models and is found in higher liver levels in those with cirrhosis and alcohol-induced injury (Teillon et al., 2010; Gao et al., 2015; Yang et al., 2017; Amir et al., 2020). However, BDNF is anti-inflammatory in the brain under most circumstances, including HE (Kang et al., 2016a; Dhanda et al., 2018). Our data showed that with the intact vagus, BDNF increased with cirrhosis, paralleling prior studies, but this circuit is broken with vagotomy, where cirrhosis does not lead to BDNF increase in the liver but does in the brain. This may be a protective mechanism since the reverse profile i.e., higher liver BDNF and lower brain BDNF is found in psychiatric disorders (Yang et al., 2017).

Ultimately, it is striking that most differentiators in the setting of vagotomy compared to conventional mice only occurred after the induction of cirrhosis through CCl4. Vagotomized control mice and conventional control mice were otherwise similar in most outcomes related to the brain and liver. Therefore, despite the relatively lower dysbiosis in vagotomized control mice, they were equally susceptible to CCl4-induced cirrhosis. Gavage with CCl4 typically induces toxic cirrhosis with dysbiosis and neuro-inflammation without the diversion of bile flow (Kang et al., 2016a). These interactions were enhanced in vagotomized animals, pointing to an important role of hepatic parasympathetic innervation in not only fatty liver as previously described but also in cirrhosis (Gao et al., 2015; Amir et al., 2020). Moreover, the decoupling of the gut-liver-brain axis found due to a lower dysbiosis but higher neuro-inflammation in vagotomized cirrhosis shows that the neuronal input from the liver may be an important way station in the gut-brain communication in cirrhosis. Vagotomy-induced lower hepatic and higher BDNF cortical expression also suggest that the neurotrophic factors may have a major role in the gut-liver-brain axis in cirrhosis (Amir et al., 2020).

Our study was limited since we used isolated hepatic vagotomy and not a more radical subdiaphragmatic approach (Bercik et al., 2011). However, the latter approach affects most gastrointestinal organs and can impact acid secretion and motility, all of which can confound the results by affecting microbiota independently. We used the entire frontal cortex given its involvement in HE but further work will be necessary to characterize the cell type in which expression of these neural plasticity and inflammatory markers occurs. We only used the CCl4 model *via* gavage because, unlike the bile duct ligated model, it does not result in microbiota change due to immediate bile diversion (Fouts et al., 2012; Dhanda et al., 2018). However, it does not cause major behavioral changes in mice, which is why we used inflammatory gene expression as the readout (Butterworth et al., 2009); future behavioral testing models are needed (DeMorrow et al., 2021).

We conclude that the parasympathetic innervation of the liver modulates hepatic steatosis, neuro-inflammation and dysbiosis even after the development of cirrhosis using CCl4 gavage. These data that vagal innervation of the liver plays an important role through modulation of BDNF in the gut-liver-brain axis, which has implications for the pathogenesis of cirrhosis and associated complications.

## Data Availability Statement

Data are available now at https://www.ncbi.nlm.nih.gov/Traces/study/?acc=PRJNA735706.

## Ethics Statement

The animal study was reviewed and approved by Virginia Commonwealth University IACUC.

## Author Contributions

JB and HZ conceptualized and were involved at all levels of the study. YZ, JK, DZ, SG, YW, and YT were involved in the animal handling, sacrifice, and experiments. JG-M and PH were involved in experiments design and manuscript revision. HL was involved in histological analysis. All authors contributed to the article and approved the submitted version.

## Conflict of Interest

The authors declare that the research was conducted in the absence of any commercial or financial relationships that could be construed as a potential conflict of interest.

## Abbreviations

HE: hepatic encephalopathy
CCl4: carbon tetrachloride
BDNF: brain derived neurotrophic factor
MCP-1: monocyte chemoattractant protein-1
IL: interleukin
IBA1: ionized calcium-binding adaptor molecule 1
GFAP: glial fibrillary acidic protein
SREBP1: sterol regulatory element-binding protein 1
INSIG2: insulin induced gene 2
LEfSe: linear discriminant effect size.
